# Artisan versus Artiflex phakic intraocular lens implantation in the treatment of moderate to high myopia: meta-analysis

**DOI:** 10.1186/s12886-021-01930-6

**Published:** 2021-04-10

**Authors:** Chenting Hou, Hui Li, Jiangfeng Li, Jinjian Li, Hui Peng, Qing Wang

**Affiliations:** 1 Department of Ophthalmology, the Affiliated Hospital of Qingdao University, 16 Jiangsu Road, Qingdao, 266000 Shandong Province China; 2 Department of Ophthalmology, the First People’s Hospital of Anqing, Anqing, Anhui Province China; 3 the Affiliated Hospital of Qingdao University, Qingdao, Shandong Province China

**Keywords:** Artisan PIOL, Artiflex PIOL, Myopia, Meta-analysis

## Abstract

**Background:**

To compare the postoperative safety, efficacy, predictability, visual quality and biomechanics after implantation of Artisan vs. Artiflex phakic intraocular lenses (PIOLs).

**Methods:**

Pubmed, Embase, Cochrane Library were conducted up from January 2000 to February 2020. Comparative clinical studies reporting in accordance with the eligibility criteria were included in this meta-analysis. The pooled weighted mean differences (WMDs) and odds ratios (ORs) with corresponding 95% confidence intervals were calculated.

**Results:**

Comparative trials with myopia patients were selected in this review. The pooled WMD and OR estimates statistical significance in terms of postoperative best corrected visual acuity (BCVA), efficacy, postoperative spherical equivalence (SE), predictability, contrast sensitivity and mean intraocular higher-order aberrations (HOA) (mm) for a 6-mm pupil, manifesting that Artiflex PIOL showed evident beneficial effect for correcting myopia compared to Artisan PIOL. There was no significant difference in the incidence of complications between the two groups.

**Conclusion:**

Both of two techniques were safe and effective for myopia and compared to Artisan PIOL, Artiflex PIOL had significant improvement in efficacy, predictability, contrast sensitivityand HOA, except safety and complications in the treatment of moderate to high myopia.

**Supplementary Information:**

The online version contains supplementary material available at 10.1186/s12886-021-01930-6.

## Background

The phakic intraocular lens (pIOL) implantation has been proved to be a safer technique for correcting myopia ranging from − 6.00D to − 20.00D compared to excimer laser techniques, showing significant advantages on the maintaining of best corrected visual acuity (BCVA), contrast sensitivity (CS), biomechanical stability and visual effects [1–3]. Of various PIOLs having been designed, Artisan and Artiflex lenses, considered to provide good safety and efficacy, had been widely used since they were introduced into the market [4, 5].

Both of them are implanted in the same location and fixated directly to the iris tissue, but they differ in material properties and surgical incision size [5]. Artisan lense should be inserted through a 6.2-mm incision due to its polymethylmethacrylate (PMMA) material. Unlike the rigid version, Artiflex is a foldable lense and only require a 3.2-mm corneal incision to be implanted during the surgical process, even without sutures, potentially affording a lower incidence of surgically induced astigmatism [6]. Up to now, a number of clinical trials [4, 5, 7–15] comparing Artisan with Artiflex PIOLs for moderate to high myopia were conducted to obtain broader and clearer evidence to investigate which one was superior in terms of clinical outcomes from varying aspects. However, no summative conclusions were produced because there were no sufficient qualified studies for systematic analysis, and heterogeneity existed in the outcome indicators and follow-up duration of each study, making the results biased to some extent.

Meta-analysis is a very useful method in the medical yield, being capable of providing more reliable results than a separate experiment, particularly in analysis of the unexplained studies [16, 17]. In order to improve the accuracy and reliability of the results, phakic intraocular lens in all studies were checked to insure the same. In majority, Artisan pIOL (model 204, Ophtec BV, Groningen, the Netherlands) is an iris claw-fixated pIOL with a rigid convex-concave polymethyl methacrylate (PMMA) model with an optic of 6 mm, implanted through a 5.0–6.0 mm incision. Only in Shin et.al research, Artisan PIOL of model 206 was also used in operations. Artiflex (model 401), in all studies, with a flexible 6.0 mm poly-silicone optic and PMMA haptics, can be inserted through a 3.2 mm incision.

Therefore, the present meta-analysis was performed to compare the visual outcomes and complications between Artisan PIOL and Artiflex PIOL implantation for myopia based on the data from published relevant trials, so as to provide strong evidence-based medicine for the choice of correction strategies for moderate to high myopia.

## Materials and methods

### Study selection

A systematic retrieval was conducted to select clinical controlled trials using electronic databases- PubMed, Embase, and Cochrane Library published from January 2000 to June 2020. The following key words were used to retrieve the relevant literature: (“Artisan” OR “fixed phakic intraocular lens” OR “Artisan PIOL”) AND (“Artiflex” OR “foldable” OR “flexible phakic intraocular lens” OR “Artiflex PIOL”). Additionally, we also screened the references of the original articles to prevent the omissions for this meta-analysis. Furthermore, only studies including human subjects were included.

### Study inclusion and exclusion criteria

Eligibility Criteria: (1) study design: retrospective or prospective comparative study; language was limited to English. (2) participants: patients older than 18 years of age and refraction was stable within 1–2 years; excluding cataract, glaucoma, amblyopia, retinal detachment, retinopathy or any other medical histories of ophthalmic disease. (3) intervention: the two groups were treated with Artisan and Artiflex phakic intraocular lens respectively. (4) follow-up duration: more than 6 months.

Exclusion criteria: (1) patients without moderate to high myopia (equivalent spherical lens: > − 3.00); (2) patients who had contraindications or had undergone ophthalmic surgeries; (3) Case reports, comments, editorials, proceedings, personal communications; and studies that did not report quantitative results.

All articles that were found were carefully screened, and those that reported original clinical postoperative data were selected. Data from previously reported cases included in different articles were excluded to avoid duplication of data.

### Quality assessment

The two investigators independently assessed the methodological quality of the included studies according to the risk of bias evaluation criteria recommended by the Cochrane Manual. The assessment included the following aspects: (1) Random allocation; (2) allocation concealment; (3) blind methods implementations; (4) selective reporting; (5) incomplete outcome data; (6) other bias sources. Each criterion was assessed as “low risk of bias,” “unclear,” or “high risk of bias.” Studies that fully met the above criteria indicated that the risk of bias was the lowest, and the quality level was “A”; if the evaluation results partially met the above criteria, the probability of bias was moderate, and “B” was the quality rating; and those did not meet the above criteria at all, stating that the likehood of bias was extremely high and had a quality level of “C.” Any disputation over evaluation results was resolved by focused discussion.

### Data extraction

Data collection was independently conducted by two researchers using electronic database. When there was uncertainty about whether the articles were qualified or not, we discussed to resolve the discrepancies and make the final decision. A customized data extraction form, described in the Cochrane Handbook for Systematic Reviews of Interventions, was used to record the first authors of all studies, the published year of the study, country, the intervention design, the number of patients, patients’ age and gender, follow-up duration, and major outcomes.

### Outcome measure

We specified safety index (ratio postoperative BCVA/preoperative BCVA), efficacy index (the ratio of postoperative UCVA to preoperative BCVA) and predictability (proportion of eyes of refractive spherical equivalent [SE] within ±1.0 diopters [D]) at 1 year post-operation as primary visual outcomes. Intraocular pressure, intraocular higher order aberrations (HOAs) for 6 mm pupil diameters, contrast sensitivity (CS) log value at 3, 6, 12 and 18 cycles per degree (cpd) and complications were regarded as secondary visual outcomes. When data at 1 year were not reported, the outcomes at the follow-up time point closest to 1 year were replaced.

### Statistical analysis

Quantitative primary and secondary outcome data were inputted and analyzed respectively using Stata version 16.0 (Stata Corp LP, College Station, TX). Random-effects models were used to calculate and analyze the continuous and dichotomous variables of outcomes. For continuous variables, means and standard deviations were used to calculate weighted mean differences (WMDs) with 95% confidence intervals (CIs). For binary outcomes, relative effect sizes were calculated as odds ratios (ORs) with 95% CIs. As for positive results (efficacy, predictability and CS are positive indicators, because a higher value suggests a better result), WMD > 0 or ORs > 1 correspond to beneficial effects of the former method in contrast to the latter method. As for negative results (safety and HOAs are negative indicators, because a higher value suggests a worse result), ORs < 1 or WMD < 0 correspond to beneficial effects of the former method in contrast to the latter method. Statistical heterogeneity among researches was detected using I^2^ statistics, and I^2^ > 50% was considered to have significant heterogeneity. When significant heterogeneity appeared, sensitivity analysis or subgroup analysis was performed to exclude studies with great differences or studies with high risk of bias to detect the stability of the combined effect results. Because there were too few studies reporting certain outcome to be suitable for subgroup analysis, sensitivity analysis was conducted to detect the stability of the conclusions. A *P* value less than 0.05 was regarded statistically significant.

## Results

### Study screening

**Fig. 1:**
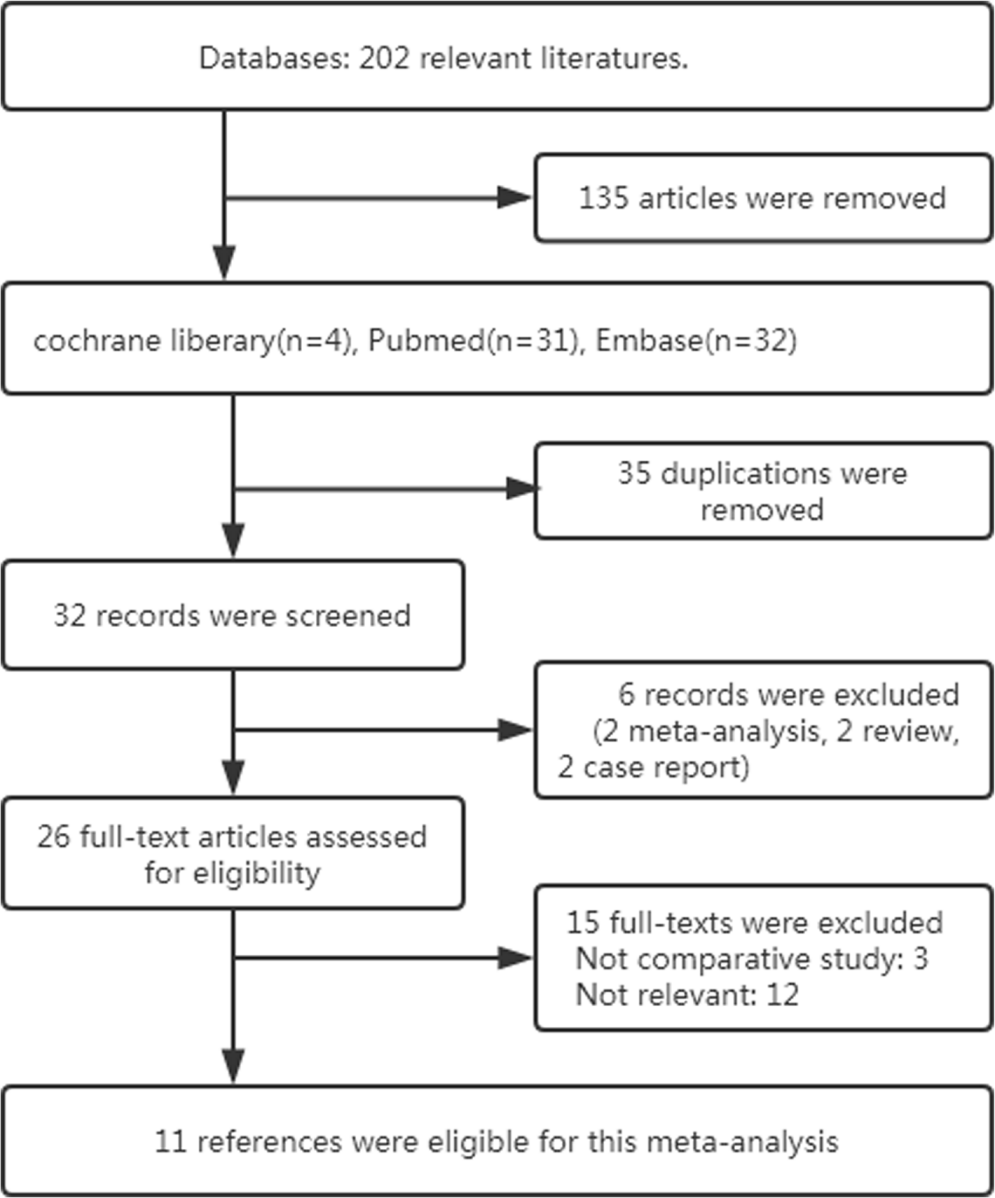
Flowchart for the studies analysis

Figure 1 presents the summarized flowchart for the study screening procedure. The literature search yielded 202 potentially relevant articles. Of these, 32 articles were retrieved from electric database after selecting the titles and abstracts. After taking out 21 studies on the basis of the pre-defined inclusion criteria, 11 studies that met the eligibility criteria were included in this meta-analysis.

### Study characteristics and quality

A summarized characteristics of all including studies are presented in Tables 1 and 2.

**Table 1:** Basic characteristics of included trials

	Country	type	Age		Sex(F/M)	
			Artisan	Artiflex	Artisan	Artiflex
Hedayatfar et.al(2017) [7]	Iran	Prospective	25.5 ± 6.0	27.8 ± 4.8	1.29	2.28
Parsipour et.al(2016) [4]	Iran	Prospective	NR	NR	NR	NR
Aerts et.al(2015) [8]	Netherland	Retrospective	51.2 ± 10.1	45.5 ± 11.3	238/133	171/86
Karimian et.al(2014) [9]	Iran	Historical cohort	27 ± 3	30 ± 5	NR	NR
Alio et.al(2013) [10]	Spain	Retrospective	32 ± 11.46	36.81 ± 8.28	NR	NR
Shin et.al.(2013) [11]	Korea	Retrospective	31.75 ± 8.30	31.47 ± 6.39	32/8	27/9
Torri et.al (2013) [12]	Japan	Retrospective	39.2 ± 8.1	37.6 ± 7.2	NR	NR
Peris-Martinez et.al(2009) [13]	Spain	Retrospective	30 ± 5	33 ± 6	NR	NR
Kohnen et.al(2008) [14]	USA	Retrospective	32 ± 10	34 ± 10	7/2	7/1
Tahzib et.al(2008) [15]	USA	Retrospective	40.0 ± 12.0	41.0 ± 7.8	8/19	13/9
Coullet et.al (2006) [5]	France	Double-blind	37.8 ± 9	37.8 ± 9	NR	NR

**Table 2:** Basic characteristics of included trials

	Eyes		Follow-up		pre SE	
	Artisan	Artiflex	Artisan	Artiflex	Artisan	Artiflex
Hedayatfar et.al(2017) [7]	16	56	1w,1 m,3 m,6 m,12 m		−16.64 ± 6.83	−10.33 ± 3.15
Parsipour et.al(2016) [4]	24	33	12 m		−10.39 ± 8.43	− 10.39 ± 2.29
Aerts et.al(2015) [8]	371	257	17 ± 7.2(6 m, 1y, 2y)		−12.7 ± 5.0	−9.29 ± 2.8
Karimian et.al(2014) [9]	40	36	30 ± 11 m		−11.6 ± 3.7	−9.59 ± 1.97
Alio et.al(2013) [10]	16	15	NR		−13.38 ± 4.33	−11.32 ± 3.10
Shin et.al.(2013) [11]	40	36	3 m		−9.88 ± 2.19	−7.67 ± 1.92
Torri et.al (2013) [12]	23	30	6 m		−11.84 ± 4.9	− 9.78 ± 3.2
Peris-Martinez et.al(2009) [13]	12	18	12 m		−9.2 ± 2.6	− 9.6 ± 2.6
Kohnen et.al(2008) [14]	15	15	6, 12 m		−10.23 ± 1.92	−8.78 ± 1.56
Tahzib et.al(2008) [15]	22	27	1w, 1 m, 3 m, 12 m		−9.9 ± 2.74	−9.95 ± 1.43
Coullet et.al (2006) [5]	31	31	12 m		−10.3 ± 3.2	−9.5 ± 2.2

Footnotes: *F/M* means female/male, *Pre SE* means spherical equivalent before surgery, *NR* not reported in this study

Selected trials were published from 2000 to 2020. Overall, the number of participants was in the range of 30 to 628 in both PIOL groups of the studies. The number of all patients meeting the criteria was 1164 (610 participants treated with Artisan PIOL, and 554 participants treated with Artiflex PIOL). For experimental type, the analysis included 8 retrospective studies, 2 prospective studies and 1 historical cohort study. 10 studies used lens with Artisan model 204, Artisan model 204 and 206 were both used in Shin et.al. study.

The risk of bias obtained by the Cochrane risk assessment is shown in Table 3.

**Table 3:** Quality assessment of included studies

Included in research	Random Allocation (selection bias)	Allocation concealment (selection bias)	Blinding of participants and personal (implemention bias)	Blinding of outcome assessment (measurement bias)	Selective reporting (reporting bias)	Incomplete outcome data (follow-up bias)	Other bias	Quality level
Hedayatfar et.al	H	U	U	U	L	L	L	B
Parsipour et.al	H	U	U	U	L	L	L	B
Aerts et.al	H	U	U	U	L	H	L	B
Karimian et.al	H	U	U	U	L	L	L	B
Alio et.al	H	U	U	U	L	L	L	B
Shin et.al.	H	U	U	U	L	L	L	B
Torri et.al	H	U	U	U	L	L	L	B
Peris-Martinez et.al	H	U	U	U	L	L	L	B
Kohnen et.al	H	U	U	U	L	L	L	B
Tahzib et.al	H	U	U	U	L	L	L	B
Collet et.al	H	L	L	L	L	L	L	A

*H* high risk of bias, *U* unclear, *L* low risk of bias

Reporting bias and other biases related to randomization of all studies were judged to be low-risk; Selection bias, implementation bias and measurement bias related to allocation hiding were considered to be uncertain risk for the majority because these three items were not reported in the most of the studies. One trial conducted by Coullet et.al [5] used double-blind, thus, gained low risk. In terms of the follow-up bias, only Aerts et.al [8] reported incomplete outcome data so as to be judged as high risk of bias.

### Meta-analysis outcomes

#### Primary outcomes

##### Postoperative BCVA

Four studies [4, 9, 11, 15] measured mean postoperative BCVA. The difference of BCVA between two groups was statistically significant (Pooled WMD: 0.072; 95% CI: 0.005 to 0.139; *P* = 0.035) (Additional file 1: Appendix I).

##### Safety and efficacy

**Fig. 2:**
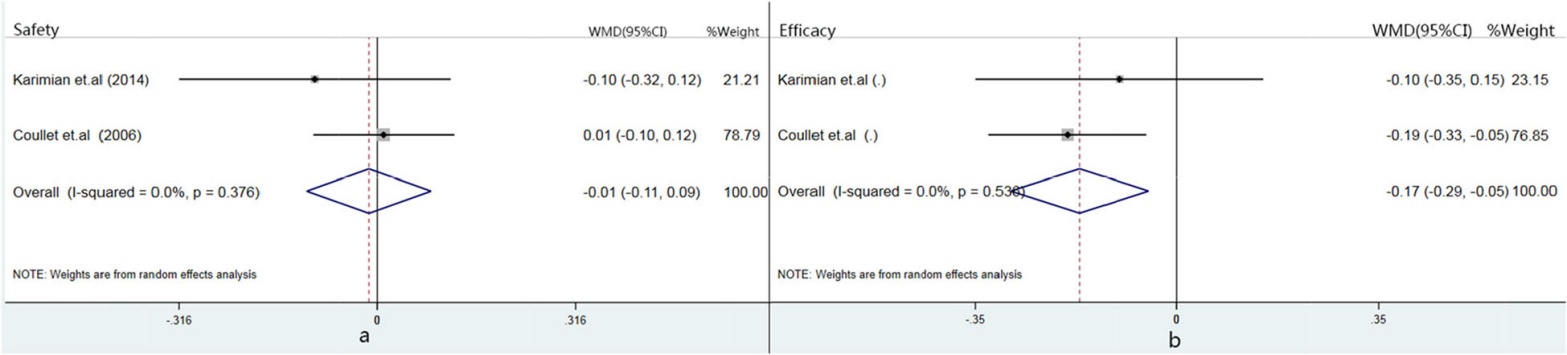
**a** shows forest plot of safety index; **b** shows forest plot of efficacy index

Figure 2a showed Artisan PIOL and Artiflex PIOL were similar in terms of 1 year postoperative safety. As for Efficacy, Artiflex PIOL obviously had higher value than Artisan PIOL group (pooled WMD: -0.169; 95CI: − 0.289 to − 0.049; *p* = 0.006, Fig. 2b).

##### Predictability (postoperative SE)

The data of postoperative SE were available in 7 studies, 5 of which were followed up for 1 year, and the other two for 3 and 6 months, respectively. Five studies with 1 year follow-up were calculated in this analysis, showing that the WMD of mean postoperative SE 1 year after surgery was favorable to Artiflex PIOL over Artisan PIOL (Pooled WMD: -0.249; 95% CI: − 0.44 to − 0.058, *p* = 0.01). In addition, Table 4 presents the combined effect of three studies showing proportion of eyes with postoperative SE within ±1.0 D of the patients, revealing that Artiflex PIOL led to better predictability compared with Artisan PIOL (pooled OR: 0.254; 95%CI: 0.126 to 0.511, *p* < 0.01).

**Table 4:** WMD of SE and Odds Ratio of proportion of eyes with SE within ±1.0 D 1 year after operation

Study (publication year)	Follow-up	SE value		SE within ±1D	
		WMD [95% CI]	%Weight	OR[95% CI]	%Weight
Parsipour et.al(2016) [4]	1y	−0.39[− 0.974,0.194]	8.48	0.378 [0.122,1.172]	38.33
Karimian et.al(2014) [9]	30 ± 11	−0.38[− 0.587,-0.173]	27.78	0.136 [0.036,0.521]	27.27
Tahzib et.al(2008) [15]	1y	0.02[−0.221,0.261]	24.88	/	/
Kohnen et.al(2008) [14]	1y	−0.16[− 0.479,0.159]	19.16	/	/
Coullet et.al(2006) [5]	1y	−0.43[− 0.741,-0.119]	19.70	0.266 [0.081,0.879]	34.39
Combined effect size		Pooled WMD[95% CI]: −0.249[− 0.44,-0.058], Test of WMD = 0: z = 2.56, p = 0.01*		Pooled OR[95% CI] 0.254 [0.126,0.511], Test of OR = 1: z = 3.84, *p* < 0.01 *	
Heterogeneity		chi^2^ = 8.15(d.f. = 4), I^2^ = 50.9%, *p* = 0.086		Chi^2^ = 1.32(d.f. = 2), *p* = 0.517, I^2^ = 0	

*SE* spherical equivalent

##### Intraocular pressure

Five studies mentioned postoperative IOP. There was no significant difference on IOP between Artisan PIOL and Artiflex PIOL (pooled WMD: -0.434; 95% CI: − 1.099 to 0.231; *p* = 0.20) and no significant heterogeneity (I^2^ = 15.8%, *P* = 0.314) (Additional file 1: Appendix II).

##### High-order aberrations

**Fig. 3:**
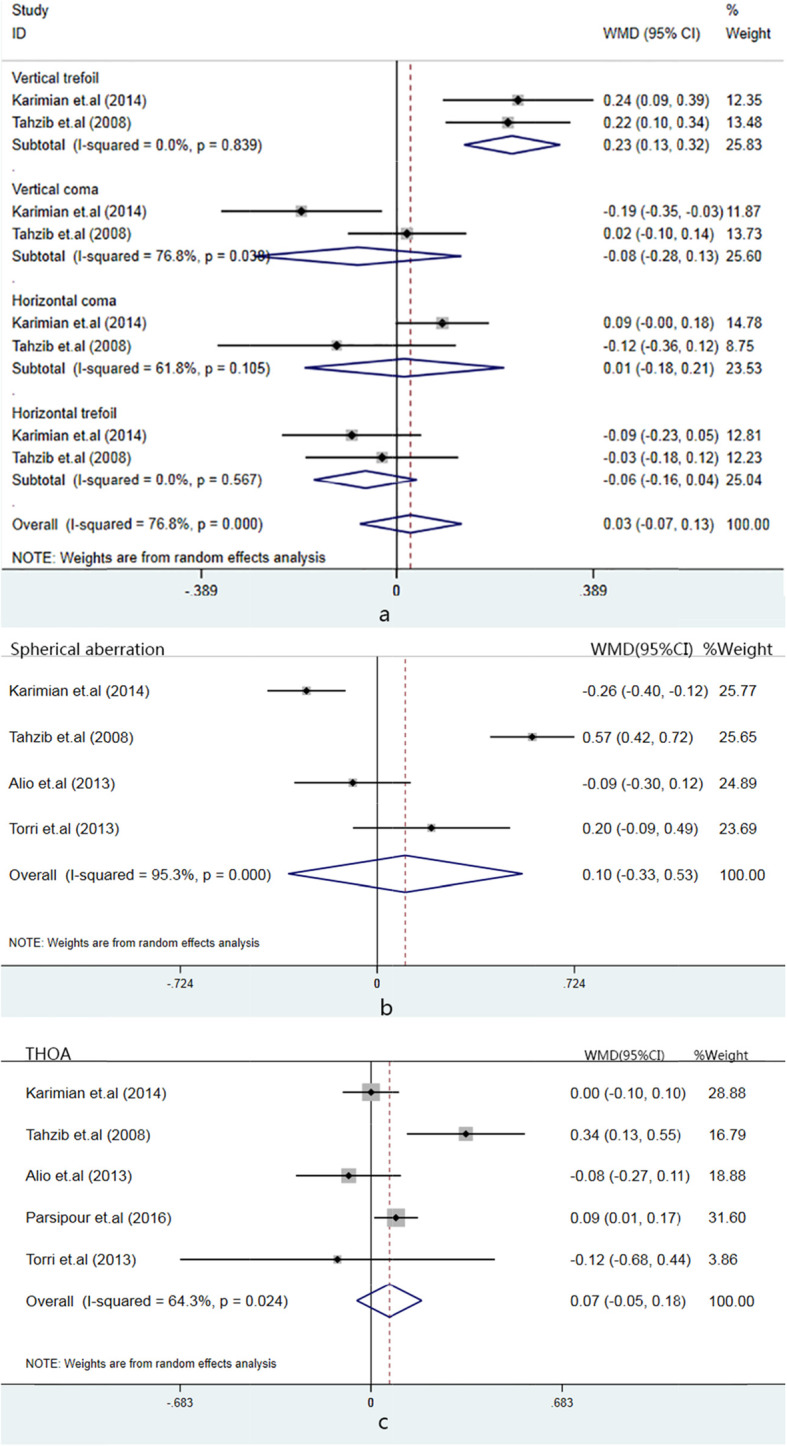
Forest plot of the intraocular high-order aberrations. **a** shows forest plots of vertical trefoil, vertical coma, horizontal coma, horizontal trefoil; **b** shows forest plot of spherical aberration; **c** shows forest plot of total high-order aberration

Intraocular high-order aberrations with optical zone of 6-mm diameters were measured in some trials. The difference of postoperative vertical trefoil was statistically significant between Artisan and Artiflex PIOL (Pooled WMD: 0.228; 95% CI: 0.1333 to 0.323; *p* < 0.01). There was no significant difference in the vertical coma, horizontal trefoil, horizontal coma, spherical aberrations and total HOA between two techniques (Fig. 3).

##### Contrast sensitivity

The forest plot (Additional file 1: Appendix III) showed the WMD (95%CI) and I^2^ value of two eligible studies recording CS at 3 cpd (WMD:-0.1; 95%CI:-0.23 to 0.02, *p* = 0.48), 6 cpd (WMD:-0.23; 95%CI: − 0.35 to − 0.11; *p* = 0.32), 12 cpd (WMD:-0.17; 95%CI: − 0.28 to − 0.06, *p* = 0.70), 18 cpd (WMD: -0.21, 95%CI: − 0.32 to − 0.10, *p* = 0.75). There was no great difference in CS at 3pd between two groups (*p* = 0.11). The differences of WMD WMD in the Artiflex PIOL was significantly higher than that in Artisan PIOL in CS at 6 cpd (*p* < 0.01), 12 cpd (*p* = 0.003), 18 cpd (p < 0.01).

##### Complications

Seven studies did not state clearly whether any complications occurred after implanting PIOLs. Collet et.al [5] and Torri et.al [12] said no complications appeared during the follow-up period. Karimian et.al [9] reported local iris atrophy appeared after either Artisan or Artiflex PIOL implantation, 2 eyes (5%) and 4 eyes (11.1%) prospectively. Hedayatfar et.al [7] showed some patients developed uveitis, 1eye (6.25%) in Artisan group and 2 eyes (3.58%) in Artiflex group. The pooled WMD presented no significant difference between two surgical methods (Additional file 1: Appendix IV).

##### Sensitivity and subgroup analysis

As described above, significant heterogeneity in certain results was detected, so a sensitivity analysis was conducted by removing studies with the most diversity in meta-analyses. For postoperative BCVA, we carried out sensitivity analysis for the significant heterogeneity (I^2^ = 91.5%). According to the picture of sensitivity analysis (Additional file 1: Appendix V), Shin et.al [11] and Tahzib et.al [15] had greater difference than others. So we took out these two trials prospectively to get effect results. When the Shin et.al [11] study was eliminated, result remained unchanged and heterogeneity decreased lightly but still exist. This can indicate the stability of results. When the Tahzib et.al [15] study was taken out, combined effect showed the two groups weren’t different (*p* = 0.125), which was not consistent with results before excluding. But heterogeneity was still high.

**Fig. 4:**
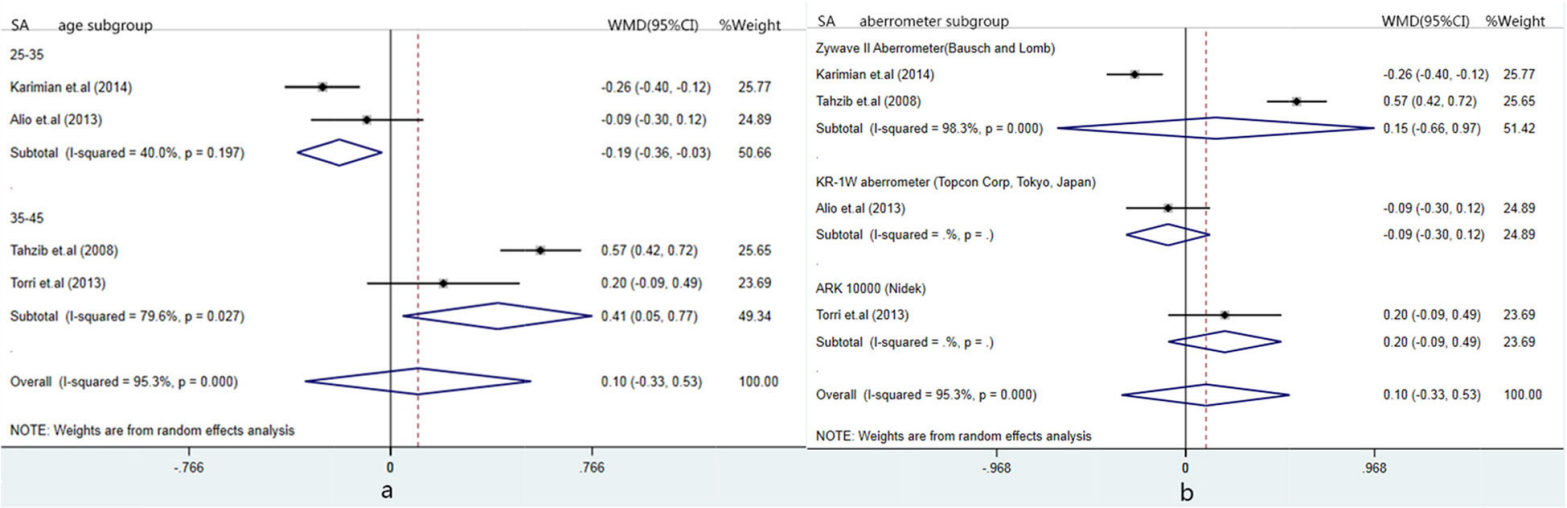
Forest plots of the subgroup analysis for spherical aberration (SA). **a** shows subgroup plot divided by age; **b** shows subgroup plot divided by aberrometer type

**Fig. 5:**
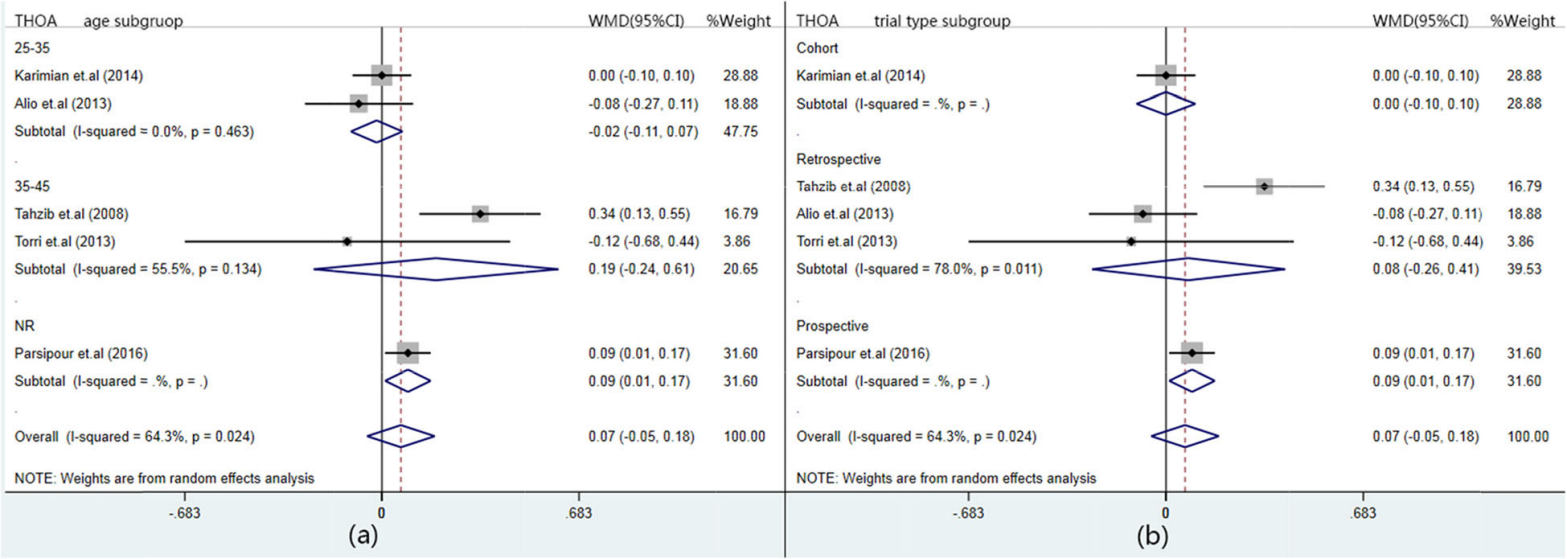
Forest plots of subgroup analysis for total high-order aberration (THOA). **a** shows subgroup plot divided by age; **b** shows subgroup plot divided by trial type

Due to the high heterogeneity in SA and THOA, sensitivity analysis and subgroup analysis were performed. As for SA, a great difference between Karimian et.al [9] and Tahzib et al. [15] can be seen from the figure (Additional file 1: Appendix VIA). Thus, these two studies were exclude prospectively to explore heterogeneous sourse. When excluding Karimian et al. [9] or Tahzib et al. [15], heterogeneity basically didn’t change with same combined result; thus, other two studies continued to be excluded one after another, the unchanged results were still heterogeneous. (Table 5) Moreover, subgroup divided by age and aberrometer type were performed. It can be found from two forest plots (Fig. 4) that there still be heterogeneity in subgroup, meaning that the results were independent of age and measurement tools. As for THOA, no great difference was found in Additional file 1: Appendix VIB. Thus, subgroup analysis based on age and experimental type was carried out and heterogeneity still exist in each subgroup (Fig. 5). The above results showed that the combined effects did not change significantly after one by one exclusion and subgroup analysis, manifesting that the meta-analysis was stable and reliability.

**Table 5:** Sensitivity analysis outcomes of postoperative BCVA and SA

		Combined effect size	Heterogeneity
BCVA	Exclude Shin et.al	WMD [95%CI] 0.097 (0.044,0.15), p < 0.01	I^2^ = 72.4%, *p* = 0.027
	Exclude Tahzib et.al	WMD [95%CI]0.063 (−0.017,0.143),*p* = 0.125	I^2^ = 91.8%, P < 0.01
SA	Before excluding	WMD[95%CI]: 0.104[−0.327,0.535],*P* = 0.635	Chi^2^ = 63.24(d.f. = 3) p = 0.0, I^2^ = 95.3%
	Exclude Karimian et.al	WMD[95%CI]: 0.232[−0.202,0.666],*p* = 0.295	Chi^2^ = 24.79(d.f. = 2) p = 0.0, I^2^ = 91.9%
	Exclude Tahzib et.al	WMD[95%CI]:-0.076[−0.321,0.169],*p* = 0.544	Chi^2^ = 8.18(d.f. = 2) *p* = 0.017, I^2^ = 75.5%
	Exclude Torri et.al	WMD[95%CI]:0.074[−0.467, 0.616],*p* = 0.788	Chi^2^ = 62.68(d.f. = 2) p = 0.0, I^2^ = 96.8%
	Exclude Alio et.al	WMD[95%CI]:0.169[−0.406, 0.744],*p* = 0.565	Chi^2^ = 59.79(d.f. = 2) p = 0.0, I^2^ = 96.7%

*BCVA* best corrected visual acuity, *SA* spherical aberration

## Discussion

PIOL implantation may provide better vision-related life quality, fewer complications and higher satisfaction with correction than LASIK for high myopia in the long term [18]. Artisan and Artiflex PIOL were widely used in the market among various designed lens. To our knowledge, a number of clinical comparative studies reported on outcomes after Artisan and Artiflex phakic intraocular lens have published [4, 5, 7–15]. It’s necessary to conduct integrative analysis to the postoperative effects of two surgical methods. This meta-analysis focuses on the comparison between Artisan and Artiflex phakic intraocular lens procedures for correcting myopia. The meta-analysis is more extensive in terms of the number of studies (11) and patients examined (1164 eyes), considering a larger range of results including safety, efficacy, predictability, intraocular pressure, ocular aberrations and contrast sensitivity based on outcomes from observational trials.

The pooled results demonstrated that there was no difference in safety and intraocular pressure between the Artisan and Artiflex-treated eyes. Except the higher level in vertical trefoil in Artisan group, there was no between-group difference on the other categories of aberration. Compared to Artisan PIOL group, Artiflex PIOL group had beneficial effect on postoperative BCVA, SE, efficacy, predictability and CS for moderate to high myopia.

Safety, efficacy and predictability are three crucial postoperative indicators in PIOL implantation evaluation. Wu et.al [19] found that in both rigid and foldable PIOL group, no difference in the percentage of eyes losing two or more lines of BSCVA and regarding efficacy and predictability, rigid PIOL was inferior to foldable PIOL. Our results demonstrated that Artiflex PIOL had more advantage on efficacy and two PIOLs provided the same degree of safety from the calculation of only two studies reporting safety and efficacy index. We evaluated the predictability of surgery with the proportions of eyes within ±1.00 D. Table 4 showed Ariflex PIOL could provide better predictability rather than Artisan PIOL, which was coincident with previous meta-analysis [19]. This result appeared to be due to the fact that the modification of the adjusted anterior chamber depth value attributed to a better accuracy of Artiflex power calculation [5]. The reason why Kochon et.al [14] research was removed automatically by analysis software was that it presented 100 percentage of eyes within ±1.00 D of emmetropia in both groups. Furthermore, evaluation of contrast sensitivity in mesopic condition presented no evident difference between two groups at spatial frequency of 3 cpd, but was significantly higher in Artiflex PIOL group at spatial frequencies of 6, 12 and 18 cpd. This result was similar with former studies [4, 19]. Therefore, Artiflex PIOL was determined to bring about the better visual quality in myopia correction.

There was no difference in intraocular pressure between the two groups. Due to no case appeared an acute rise in IOP after performing peripheral iridectomy, Kamian et.al [8] recommended peripheral iridectomy. Regarding HOA, 2 studies measured vertical trefoil, vertical coma, horizontal trefoil and horizontal coma separately. The data of these parameters from two studies was too limited to implement sensitivity or subgroup analysis appropriately. Vertical trefoil was higher in Artisan PIOL (*P* < 0.01) in contrast to Artiflex treatment, which was consistent with Karimian et.al [9]. This may result from the incision at 12 o’clock position in process of Artisan PIOL implantation. There was no significant difference in the vertical coma, horizontal trefoil, horizontal coma, spherical aberrations and total HOA between two PIOLs with a 6-mm pupillary diameter. For the high heterogeneity in researches measuring spherical aberration and HOA, sensitivity and subgroup analysis were performed. Unchanged results manifested that the outcomes of meta-analysis were stable, not overly affected by any specific research. Previous literatures showed some information relevant to aberrations. Meta-analysis of Wu et.al [19] obtained the conclusion that the rigid and foldable group differed significantly in intraocular HOA for 6-mm pupil. This disparity may result from different inputted entry and calculation software. Karimian et.al [9] reported spherical aberration was higher in the Artisan-treated eyes, which may contribute to different materials of this lens (i.e. PMMA) in comparison to lens in Artiflex PIOL. Tahzib et.al [15] concluded that Artiflex PIOL decreases while Artisan PIOL increases spherical aberrations due to differences in optical design of these two PIOLs. Torri et.al found intraoculer spherical aberration and THOA did not differ between Artisan and Artiflex PIOL, which is concurrent with ours.

With respect to complication, the present analysis evaluation demonstrated no great difference between the two procedures, in spite of the diversity of their crystal composition and notch sizes. Inflammation was more likely to occur in the flexible Artiflex lens rather than the rigid Artisan lens [7]. After iris-fixated surgery, the abnormal pressure on the iris between the crystalline lens and pIOL may lead to pigment dispersion [20, 21]. Incarceration of iris tissue may cause chronic iris irritation with destruction of the blood-aqueous barrier and subsequent inflammatory reaction [22]. Additionally, the regularity of the crystal surface partly determines the biocompatibility of IOLs, which plays a crucial role in inducing inflammation. Some reports have revealed the low biocompatibility of uveitis patients with silicone intraocular lens after cataract surgery [23, 24]. Scanning electron microscopy has confirmed a smooth and uniform surface feature for Artisan’s PMMA [25]. Unlike the better biocompatibility of the Artisan’s, the silicone–PMMA of Artiflex pIOLs was more likely to initiate anterior chamber inflammatory reaction, [26] increasing the incidence of pigment and non-pigment deposits after surgery [27].

Strictly adhering to the Cochrane guidelines and the methodology, we calculate the data meeting the eligibility criteria to evaluate clinical effects objectively. And we conducted sensitivity analysis and subgroup analysis when heterogeneity was obviously high to increase the robustness and validity of the results.

This meta-analysis, focusing on clinical outcome measures, availably carried out the conformity analysis of relatively homogenous comparative studies on varying follow-up durations. However, some limitations need to be further refined. For one thing, different PIOL implantation schemes were included in the study, and not all outcome indicators correspond to identical intervention durations in the meta-analysis, which may cause errors in the results. For another, although sensitivity and subgroup analysis showed the stability of the combined estimates of this study, the heterogeneity of kinds of postoperative index of eyes optical effect was not well handled. In addition, due to the partial absence of the inclusion information of the preoperative basic characteristics of the various researches, it is difficult to ensure the consistency of the preoperative situation, leading to a deviation in the pooled estimates of the effect of Artisan and Artiflex PIOL implantation.

## Conclusions

In summary, this meta-analysis offered the latest evidence and presented that both of two techniques were safe and effective for myopia and compared to Artisan PIOL, Artiflex-treated eyes had significant advantage in BCVA, efficacy, predictability, CS and HOA, except safety for moderate to high myopia. Further data were needed to prove the differences in the biocompatibility between both lenses. More high-quality multicenter researches need to be carried out and more data needs to be collected to reduce the risk of bias in order to make the clinical follow-up results and analyzing findings more accurate and reliable.

## Supplementary Information

**Additional file 1.**

## Data Availability

All data generated or analysed during this study are included in this published article [and its supplementary information files].
