# Supplementary material for: Artisan versus Artiflex phakic intraocular lens implantation in the treatment of moderate to high myopia: meta-analysis
Source: BMC Ophthalmol. 2021 Apr 10;21:171. doi: 10.1186/s12886-021-01930-6 (PMC8035774; doi:10.1186/s12886-021-01930-6)

Title page：

Title: Artisan versus Artiflex phakic intraocular lens implantation in the treatment of moderate to high myopia: Meta-analysis

Authors: Chenting Hou[1], MD; Hui Li[2], MD; Jiangfeng Li[1], MD; Jinjian Li[1], MD; Hui Peng[1], MD; Qing Wang[1], MD, PhD

Affiliations: [1]Department of Ophthalmology, the Affiliated Hospital of Qingdao University, Qingdao, Shandong Province, China

[2]Department of Ophthalmology, the First People's Hospital of Anqing, Anhui Province, China; the Affiliated Hospital of Qingdao University, Qingdao, Shandong Province, China

Supplement materials

Appendix Ⅰ. Information about BCVA of 4 studies and meta-analysis outcomes.

	Artisan			Artiflex			WMD[95%CI]	%Weight
	n	mean	SD	n	mean	SD		
Shin et.al.(2013)	40	-0.07	0.05	36	-0.07	0.04	0.00[-0.02,0.02]	27.21
Tahzib et.al(2008)	22	-0.01	0.08	27	-0.11	0.07	0.10[0.057,0.143]	25.06
Parsipour et.al(2016)	24	0.21	0.13	33	0.06	0.08	0.15[0.091,0.209]	22.95
Karimian et.al(2014)	40	0.07	0.13	36	0.02	0.06	0.05[0.005,0.095]	24.79

Combined effect size	Heterogeneity
Pooled WMD [95%CI] 0.072(0.005,0.139),p = 0.035	I^2=91.5%,p = 0.000

Appendix Ⅱ. Meta-analysis results of postoperative IOP.

Study IOP	Artisan			Artiflex			WMD[95%Conf.Interval]	%Weight
	n	mean	SD	n	mean	SD		
Karimian et.al(2014)	40	13	2	36	14	2	-1.00 [-1.901,-0.099]	37.77
Torri et.al(2013)	23	13.7	2.3	30	13.3	3.1	0.40 [-1.054,1.854]	17.87
Coullet et.al (2006)	31	14.4	2.4	31	14.2	2.8	0.20 [-1.098,1.498]	21.62
Tahzib et.al(2008)	22	15.5	2.71	27	16.76	3.36	-1.26 [-2.96,0.44]	13.61
Peris-Martinezet.al(2009)	12	12.5	2.9	18	12.5	2.9	0.00 [-2.12,2.12]	9.12

Combined effect size : pooled WMD[95%CI] : -0.434[-1.099,0.231], Test of WMD=0 : z= 1.28 , p = 0.200

Heterogeneity : chi2=4.75(d.f.=4), I2=15.8%, P=0.314

Appendix Ⅲ: Forest plot of the contrast sensitivity at spatial frequency of 3, 6, 12, 18 cycle per degree.

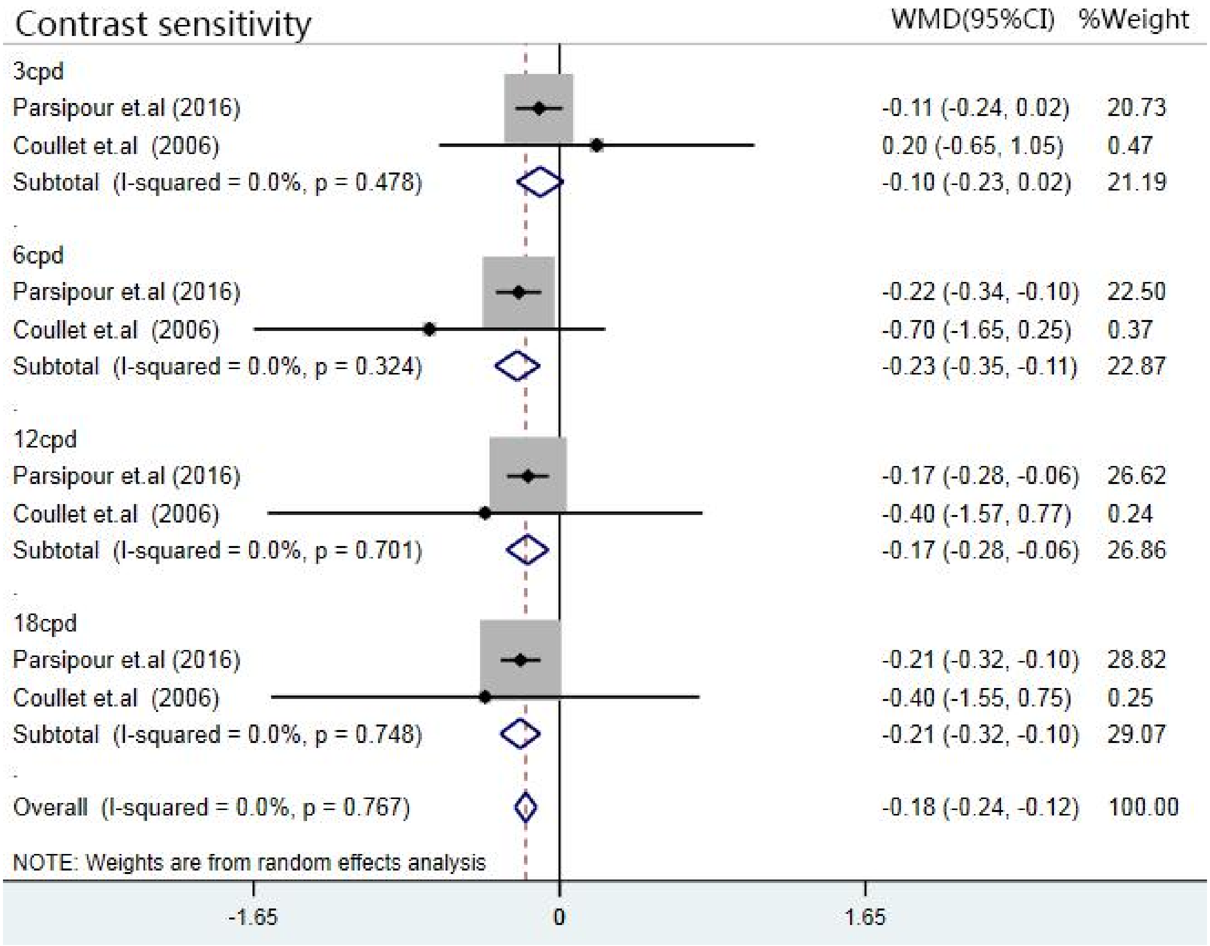

Appendix Ⅳ: Figure of forest plot for the complications

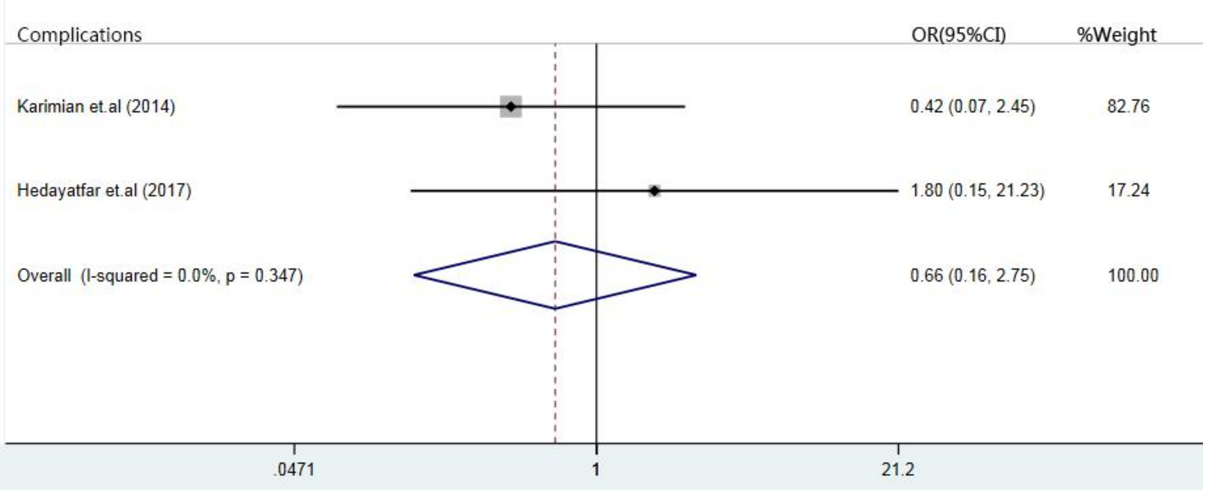

Appendix Ⅴ: Figure of sensitivity analysis of postoperative BCVA

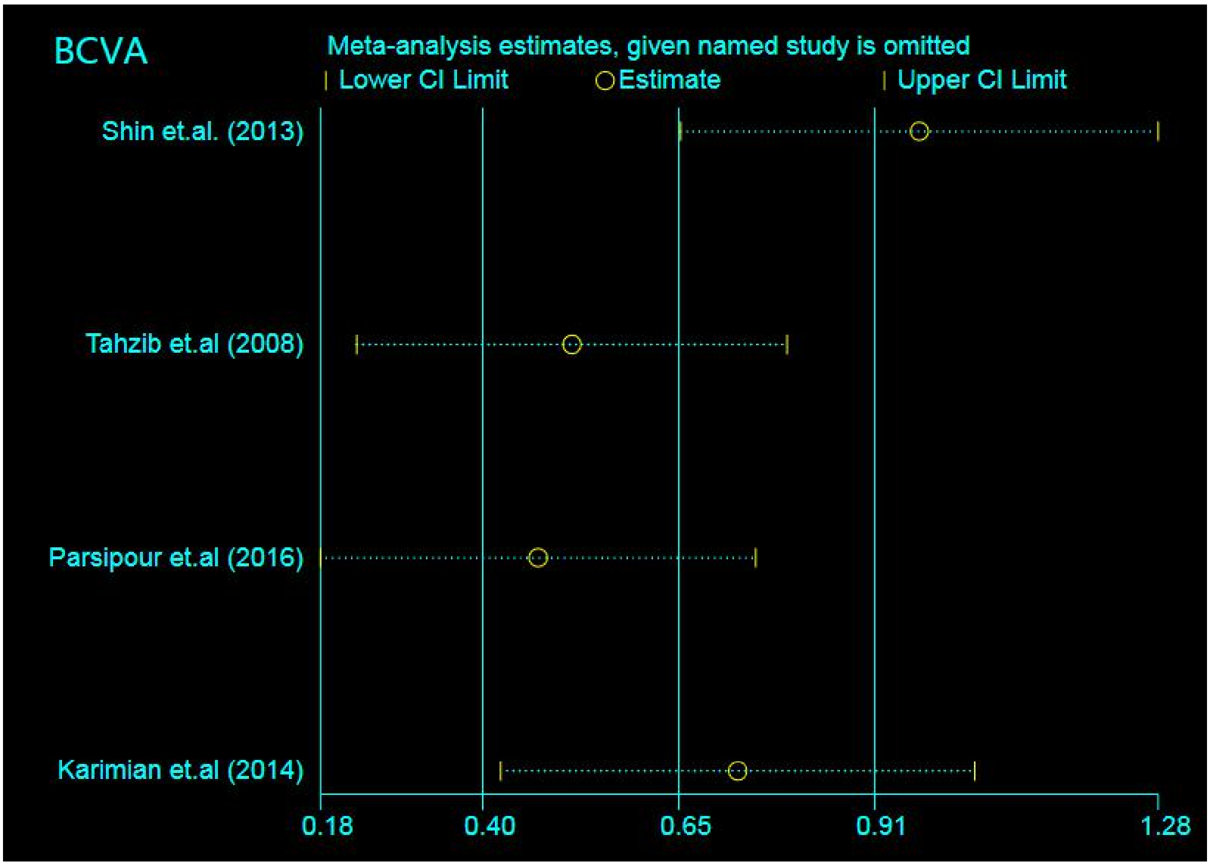

Appendix Ⅵ: Figure of sensitivity analysis for the SA and THOA, A and B, prospectively

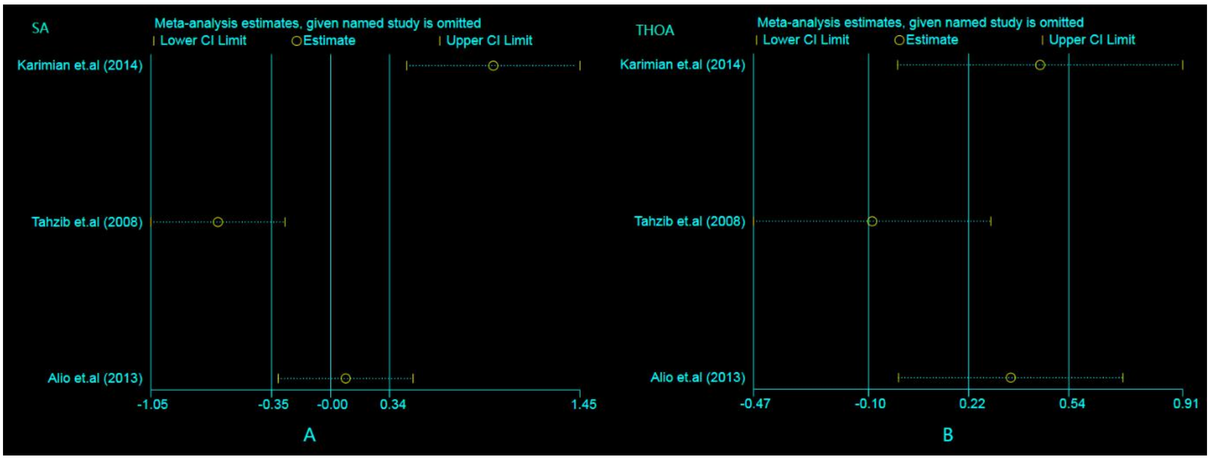

Supplement: Supplementary file 1 — Additional file 1. [file 12886_2021_1930_MOESM1_ESM.pdf]
